# The Treatment of Otorrhœa

**Published:** 1906-01-13

**Authors:** F. Faulder White


*To the Editor of* The Hospital.

### THE TREATMENT OF OTORRHŒA.

*To the Editor of* The Hospital.

Sir,—The past year has strengthened my belief in the advisability of clearing away diseased tissues from the middle ear, and that this can be satisfactorily done through the meatus has been shown by another series of successful cases, without a single failure so far as disinfection of the cavity is concerned, amongst my private cases. In some cases patients have expected greater improvement in hearing; some improvement in this respect has generally been noted. It is an interesting fact that in more than one case symptoms suggesting meningitis have disappeared after otectomy. An anonymous reviewer in the *Lancet* has said that my views are not generally accepted by the profession. The indifference often exhibited by medical men as to the treatment of running ears is, I think, the result of bad otological teaching. Only last week I operated on five cases which could not have been benefited in the least by the treatment previously ordered—the usual home syringing—for they had only pinhole perforations in the membrane. Such cases I see almost every week. That an otorrhœa lessens the expectation of life is known by every assurance society. The sufferers may be numbered by the thousand. Professional indifference to this great evil cannot surely much longer continue.

I am, yours truly,

F. Faulder White.

